# Introducing two new Main Editors of *Journal of Synchrotron Radiation*

**DOI:** 10.1107/S1600577520016550

**Published:** 2021-01-01

**Authors:** Andrew J. Allen, Yoshiyuki Amemiya, Ingolf Lindau

**Affiliations:** a National Institute of Standards and Technology, 100 Bureau Drive, Gaithersburg, MA 20899, USA; b Japan Synchrotron Radiation Research Institute (JASRI), 1-1-1 Kouto, Sayo-gun, Hyogo 679-5198, Japan; c SLAC/Stanford Linear Accelerator Center, 2575 Sand Hill Road, MS69, Menlo Park, CA 94025, USA

**Keywords:** *Journal of Synchrotron Radiation*

## Abstract

Introducing two new Main Editors of *JSR*.

Dibyendu Bhattacharyya of Bhabha Atomic Research Centre and Kristina Kvashnina of the Rossendorf Beamline – The European Synchrotron have recently been appointed as Main Editors of *Journal of Synchrotron Radiation*.

**Dibyendu Bhattacharyya** completed his undergraduation and post-graduation studies at University of Calcultta, Kolkata, India, and obtained his PhD degree from Jadavpur University, Kolkata, in 1995 based on his work at the Indian Association for the Cultivation of Science, Kolkata. After spending a few years at Newcastle Photovoltaics Applications Centre, University of Northumbria, Newcastle Upon Tyne, UK, as post-doctoral researcher, Dr Bhattacharyya joined Bhabha Atomic Research Centre, Mumbai, India, as Scientific Officer in 1997 where he is presently heading the Synchrotron Science and Multilayer Physics Section of the Atomic and Molecular Physics Division. Trained as a thin film multilayer device researcher, Dr Bhattacharyya moved to the area of synchrotron research in 2004, when the construction of India’s first hard X-ray synchrotron source Indus-2 began, and took a leading role in building up the two X-ray absorption spectroscopy beamlines. Over the years, Dr Bhattacharyya built up a highly professional group consisting of scientists, engineers and students which led to a very successful utilisation of these facilities leading to a large number of publications in reputed international journals and also contributing to product-oriented industrial research. Dr Bhattacharyya has over 260 publications in international journals and three book chapters to his credit with more than 4500 citations. He is recipient of several awards including the Homi Bhabha Scientific and Technical Excellence Award in 2014 (the highest award of Department of Atomic Energy, Government of India) and DAE-SRC Outstanding Investigator Award in 2012. Dr Bhattacharyya also holds the position of Professor in the Physical Sciences at Homi Bhabha National Institute, Mumbai, and has guided several PhD students. ▸

**Kristina Kvashnina** is ERC Group Head at the Rossendorf Beamline (BM20) of the European Synchrotron (ESRF), Grenoble, France, supported by Helmholtz-Zentrum Dresden-Rossendorf, Germany, and also Professor at the Department of Chemistry, Moscow State University, Russia. She completed her undergraduate degree in theoretical physics at Ural State University (Ekaterinburg, Russia) in 2000 and her PhD degree in experimental physics at Uppsala University (Uppsala, Sweden) in 2006, while carrying out experiments at the Advanced Light Source of Lawrence Berkeley National Laboratory, USA. She used soft X-ray absorption (XAS) and X-ray emission spectroscopy (XES) methods together with resonant inelastic X-ray scattering (RIXS) to study rare-earth containing materials. She then moved to the ESRF, where she conducted pioneering research on actinide systems using advanced high-energy-resolution X-ray spectroscopy methods (HERFD-XAS and RIXS) at the actinide *M*_4,5_ edges. In 2017, Kristina received a European Research Council (ERC) Grant to support her research on actinide and lanthanide nanomaterials aiming to better understand *f*-electron systems at the atomic level. In 2019 she received a Megagrant from the Ministry of Science and Higher Education, Russia. Kristina has published more than 140 papers in the area of X-ray spectroscopy, cited more than 2200 times, and has served as key note speaker, chair and co-chair, organizer and program committee member at numerous conferences in her area of expertise. Kristina is a member of several advisory groups and project review panels in Germany, Italy, UK, Russia, Switzerland and Hungary.

While Kristina has been active as a Co-editor of *Journal of Synchrotron Radiation* since 2019, Dibyendu is a new addition to the editorial board. The two existing Main Editors (Yoshi­yuki Amemiya and Ingolf Lindau) and Editor-in-Chief of IUCr Journals (Andrew J. Allen) are delighted to welcome Kristina and Dibyendu to the team, and look forward to working with them to further develop the journal for the synchrotron radiation and free-electron laser communities. They would also like to thank Professor Dr Ilme Schlichting (Max Planck Institute, Heidelberg, Germany) for her many years of service as a Main Editor of JSR, and wish her well as she steps down at this time.

## Figures and Tables

**Figure 1:**
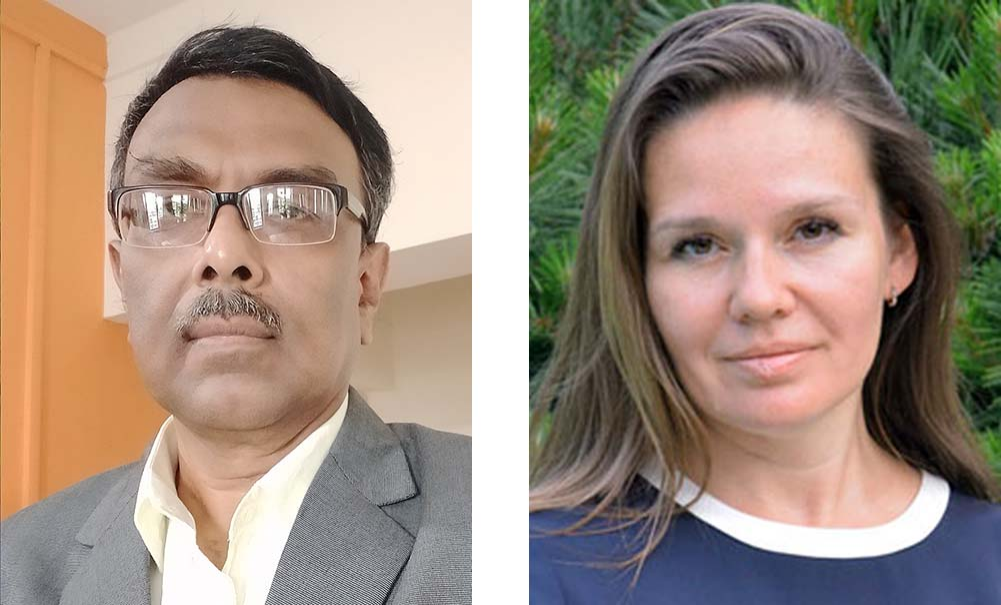
Dibyendu Bhattacharyya (left) and Kristina Kvashnina (right), new Main Editors of *Journal of Synchrotron Radiation*.

